# Fertility Preservation Needs of Men Undergoing Cancer Treatment: An Explorative Qualitative Study

**DOI:** 10.3390/curroncol33040185

**Published:** 2026-03-26

**Authors:** Marcia E. Facey, Natalie A. Baker, Anne M. Sorvari, Brittany Speller, Keith A. Jarvi, Lisa K. Hicks, Abha A. Gupta, Nancy N. Baxter

**Affiliations:** 1 Li Ka Shing Knowledge Institute, St Michael’s Hospital, Unity Health Toronto, Toronto, ON M5B 1W8, Canada; 2 Division of Urology, Department of Surgery, Mount Sinai Hospital, University of Toronto, Toronto, ON M5G 1X5, Canada; 3 Department of Surgery, Faculty of Medicine, University of Toronto, Toronto, ON M5S 1A8, Canada; 4 Division of Hematology/Oncology, St Michael’s Hospital, Toronto, ON M5B 1A6, Canada; 5 Department of Oncology, Hospital for Sick Children, Toronto, ON M5G 1E8, Canada; 6 Faculty of Medicine and Health, University of Sydney, Sydney, NSW 2050, Australia

**Keywords:** fertility preservation, men’s health, masculinity, cancer survivorship, informational needs, decision-making, qualitative research

## Abstract

Men undergoing cancer therapy are at risk of treatment-related infertility, which can negatively affect quality of life. Although fertility preservation (FP) is routinely recommended for men of reproductive age who are diagnosed with cancer, less is known about how they navigate these decisions or what informational and emotional supports they require across the diagnosis–treatment–survivorship trajectory. We conducted a qualitative secondary analysis of a subset of data from a broader study of cancer and FP decision-making. In-depth interviews with 12 men who had undergone fertility-threatening cancer treatments and had experienced FP were analyzed using a thematic analysis approach. Participants reported gaps in clear, timely and practical information, including costs, clinic access, sperm storage and future use. Men and their partners described feelings of fear, uncertainty and diminished control, especially when decisions had to be made quickly, before treatment began. Their choices were influenced not only by clinical advice but also by social expectations related to masculinity. Structural/logistical barriers such as diagnostic uncertainty, illness severity and poorly coordinated care pathways further undermined their agency and contributed to distress. These findings indicate that men’s experience of FP is not a straightforward medical procedure but a complex and ongoing process with logistical, informational and psychosocial challenges. Integrating organized counselling and continuous emotional support into oncology care may reduce distress and enhance decision-making processes.

## Simple Summary

Cancer treatments can impair fertility and affect men’s long-term quality of life. Although fertility preservation is routinely recommended for men diagnosed with cancer, less is known about how they experience these decisions or what information and emotional support they need. This study analyzed interviews with 12 men who considered or underwent fertility preservation before cancer treatment. Participants reported unmet needs for clear, timely and practical information, including how to access fertility clinics, sperm storage, future use and associated costs. They described emotional distress, uncertainty and reduced feelings of control, particularly when decisions had to be made quickly, before treatment began. Their choices were shaped not only by clinical guidance but also by social expectations related to masculinity. Partners also experienced emotional strain. These findings suggest that fertility preservation for men with cancer is a complex and ongoing process that involves both informational and emotional challenges. Integrating clear, structured communication and ongoing emotional support into oncology care may help men make more confident decisions and reduce distress.

## 1. Introduction

Men undergoing cancer treatment face significant risks to their fertility [1,2,3] and to their emotional well-being as it relates to fertility [4,5,6,7]. Infertility is both a biological and a psychosocial challenge that can threaten one’s sense of self, sexuality, body image, self-esteem, intimate relationships and life plans [8,9,10]. Infertility management is therefore a concern for individuals of reproductive age [11]. Providing timely, adequate informational and emotional supports to help men make informed decisions about fertility preservation (FP) remains an ongoing challenge for patients, clinicians and policy makers [5,12,13]. Fertility has become increasingly recognized as a key element of survivorship. As survivorship rates continue to rise, there is heightened emphasis on how survivors’ quality of life, including their potential to have children, affects overall well-being [14,15,16]. Initiatives to avoid or minimize psychological distress, which is already prevalent among cancer patients [17], and to enhance post-treatment quality of life have prompted professional bodies such as ASCO [18] and ESMO [19] to develop clinical guidelines that emphasize discussions of infertility risks and access to FP. There is also continued advocacy not only for timely and comprehensive communication about possible infertility risks associated with gonadotoxic therapies but also for making pre-treatment FP a standard component of cancer care, even for patients who are considered at low risk [20]. 

Despite existing guidelines, studies consistently show that cancer patients who are at risk of infertility frequently do not receive adequate information about FP [1,4,13,21,22,23,24,25,26,27,28,29]. Although men, with exceptions such as findings by Ussher and Perz [5], are more likely than women to be encouraged to pursue FP [23,30,31,32,33], they still face gaps in both informational and social support needs, particularly related to structured counselling and psychosocial support [10]. Patel et al. [34] reported that only 32% of male patients received counselling prior to treatment, a trend echoed in more recent research that identifies persistent social and structural barriers [20,27,29,35]. Furthermore, research indicates that there are frequent discrepancies between what patients expect or believe they should receive and the actual information provided, which suggests larger challenges in FP communication [20,28,36]. 

The assumption that male FP is a relatively straightforward clinical process and therefore requires only minimal discussion [37] may contribute to these unmet needs due to brief, superficial or inconsistent counselling. This perception of procedural simplicity, combined with counselling models that have historically centred female experiences, may inadvertently contribute to underemphasizing men’s specific concerns [38]. Although male FP is technically less complex than female FP, male cancer patients nonetheless encounter distinct emotional, informational and support-related challenges. Research suggests they may experience heightened anticipatory anxiety and psychological distress related to fears of infertility and social stigma associated with perceived impaired masculinity. However, less is understood about how culturally embedded norms of masculinity shape men’s experiences of FP decision-making and support-seeking within oncology care. Additionally, misconceptions about FP may exacerbate psychological and emotional distress [10,38]. 

Men’s experiences of cancer and FP have received comparatively limited empirical and conceptual attention, particularly in relation to their unmet informational and emotional support needs, or how masculine norms shape their decisions. Existing research is disproportionately skewed toward female experiences, and to a lesser extent, to mixed-gender cohorts. As such, it risks under-representing male-specific psychological and social distress [10,39] and limiting the possibilities for development of policies and practices that are responsive to men’s needs across the cancer trajectory. While prior studies implicitly or explicitly point to systemic and communicative challenges, there is still limited understanding of the nature of men’s FP counselling—its adequacy or inadequacy, information provision and psychosocial support in the context of cancer and potential infertility. In particular, the different ways that support gaps, structural constraints and relational dynamics intersect to shape men’s experience of FP decision-making remain underexplored. Moreover, few qualitative studies have examined how informational and emotional support needs evolve across the FP trajectory in oncology care. Addressing these gaps is critical as the absence of such care may compromise men’s ability to make empowered FP decisions, contribute to psychological distress and reinforce disparities in survivorship care. This study seeks to address this knowledge-understanding gap. Using data from a larger qualitative research study on cancer and FP [35,40,41,42,43], we examine the question, what types of information and emotional support do men need as they navigate the FP process and what challenges and complexities arise in meeting these needs, including any social or cultural influences on decision-making?

## 2. Methods

### 2.1. Study Design

We used qualitative secondary analysis [44,45] of a subset of data from a larger study that examined both men’s and women’s experiences of cancer. We focused on their diagnosis, treatment and FP decision-making, as well as the perspectives of clinicians involved in cancer care and FP. The goal of the original study was to create decision aids for cancer patients who were at risk of infertility. For the current study, we revisited the data to consider the specific question of men’s unmet support needs vis-à-vis FP. Following Thorne’s [46] guidance to meaningfully contribute to insight, in this case, of men’s support needs in the context of cancer and FP, our analysis aimed to generate a nuanced understanding of these needs.

### 2.2. Sampling and Data Collection for Original Study

We used purposive and snowball sampling strategies to recruit participants who faced onco-fertility decisions before cancer treatments. Eligibility criteria included participants (>age 18) who had completed fertility-threatening cancer treatments for any malignancy within the previous 5 years. Individuals with recurrent cancer were excluded. We aimed for maximum variation in our sample by using multiple recruitment strategies: (i) physicians identified eligible patients from their practices. The initial contact with potential participants was via a standardized invitation letter; (ii) we recruited through nationally known patient advocacy organizations such as Wellspring Cancer Support, Lymphoma Canada, Leukemia and Lymphoma Society of Canada, Survive and Thrive and Cancer Fight Club; and (iii) snowball sampling.

A PhD-trained qualitative methodologist, located in Ontario, Canada, conducted semi-structured telephone interviews between 2020 and 2022. Interviews ranged from 45 min to 2 h. We used an interview guide that was pilot-tested and modified iteratively. Topic areas included (i) FP discussions—when they happened, with whom, who initiated, types of information received, and on reflection what was missing; (ii) FP decision-making processes—fertility referrals/consultations, available decision-making time, and types of supports with decision-making.

### 2.3. Data Analysis

We used a thematic analysis approach and an interpretive lens perspective [47,48] to inductively and iteratively code transcripts and to identify and categorize patterns across the men’s accounts. Analysis focused on segments of data relevant to their experiences of FP and the nature and types of supports received or required. Initial codes (e.g., ‘FP referrals and discussions’, ‘timing of information’, ‘making decisions’ and ‘being at the clinic’) captured features relevant to informational and support needs. Codes were refined through close and repeated readings of transcripts, comparisons across the transcripts and through ongoing team discussions. Interpretive differences were resolved through discussions, with sensitivity to how our disciplinary backgrounds and assumptions might influence our understanding of the data. Through this approach, we identified two main categories of support needs: informational and emotional. In the subsequent analysis phase, we examined contextual factors that facilitated or constrained the men’s access to FP and supports. This interpretative process moved us toward explanations of the complexities underlying how and why support needs were met or remained unmet and how decisions were affected.

### 2.4. Ensuring Rigour

To enhance the trustworthiness of the study [49], all interviews were audio recorded, transcribed verbatim and audited to ensure transcription accuracy [50]. Analytic rigour was supported through iterative coding, reflexive memoing and ongoing discussions among our multidisciplinary team members. Transcripts were read multiple times and detailed summaries and memos that described the data and emerging codes, concepts and ideas were produced. These summaries and memos were further analyzed and consolidated into one document, which formed the basis for further team discussions. These discussions provided an additional level of analysis and enhanced analytic credibility. They provided opportunities to compare and challenge interpretations, examine alternative explanations and ensured concepts and categories reflected the breadth and depth of the data. A codebook was maintained to document evolving definitions and to facilitate exploration of the various interpretations of the codes by the team members [48]. NVivo (Version 12, QSR International) was used to organize data and to facilitate analysis. Ethical approval was obtained from the institutional Research Ethics Board (#18-237). Consent forms were read to participants at the start of the telephone interviews. All participants gave verbal consent to participate and agreed to be contacted in the future if needed.

## 3. Results

**Figure 1:**
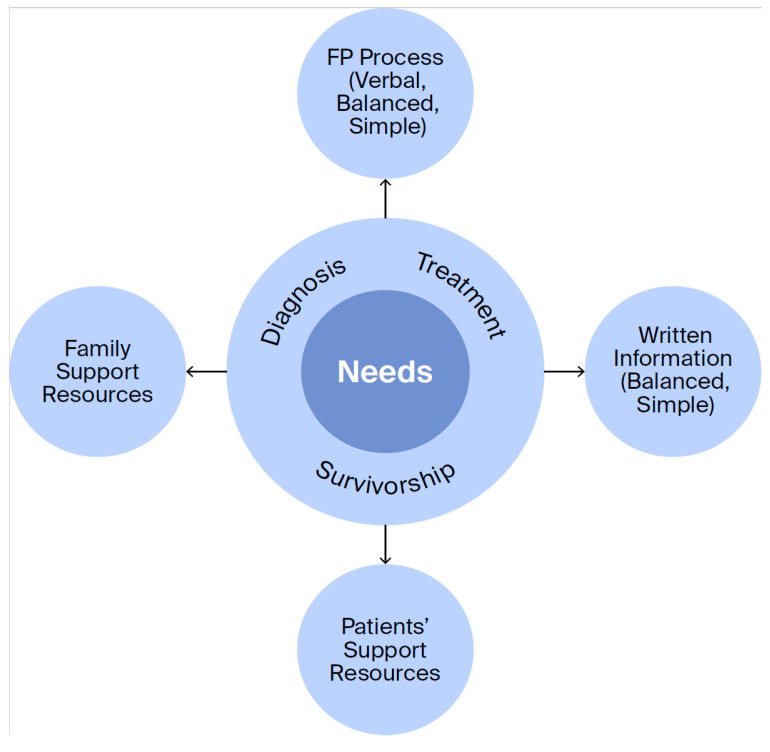
Informational and emotional needs across the FP-cancer care trajectory.

The final sample comprised 12 men who faced decisions about fertility preservation prior to starting cancer treatments. The average age at diagnosis was 31 years. Nine participants were diagnosed with testicular cancer and three with lymphoma. Eight men chose to pursue FP and four did not (See Table 1). On average, the men were interviewed two years after diagnosis. In the following sections we describe the unmet informational and emotional support needs across the diagnosis–treatment–survivorship continuum (Figure 1, Table 2 and Table 3). We then discuss the challenges or complexities of meeting these needs.

### 3.1. Informational Support Needs

Informational support needs refer to the essential verbal and written details that helped the men understand FP and guide their decision-making (Table 2). It includes information on where and how to access psychosocial supports, such as counselling, to help them manage their experiences of both cancer and FP. The men’s desire for information was illustrated in the comment of one participant who said, ‘It’s hard when you don’t know what the questions are’, and by another who described the FP process as ‘stressful and uncomfortable’ and likened it to ‘walking down a hallway blindfolded [because] nobody knew how to do it’ (P2a). Although most of the men recalled being advised or encouraged to do FP (‘everybody told me to do it’), they characterized these exchanges as brief and mostly verbal. Information provided at the point of diagnosis was described as ‘scant’, ‘very light’ or ‘bare minimum’. One man recalled that his discussion with his oncologist was not really a discussion, but a caution about chemotherapy-induced infertility and a suggestion that he ‘should really, really, really do it’ to prevent future regret (P1).

**Figure 2:**
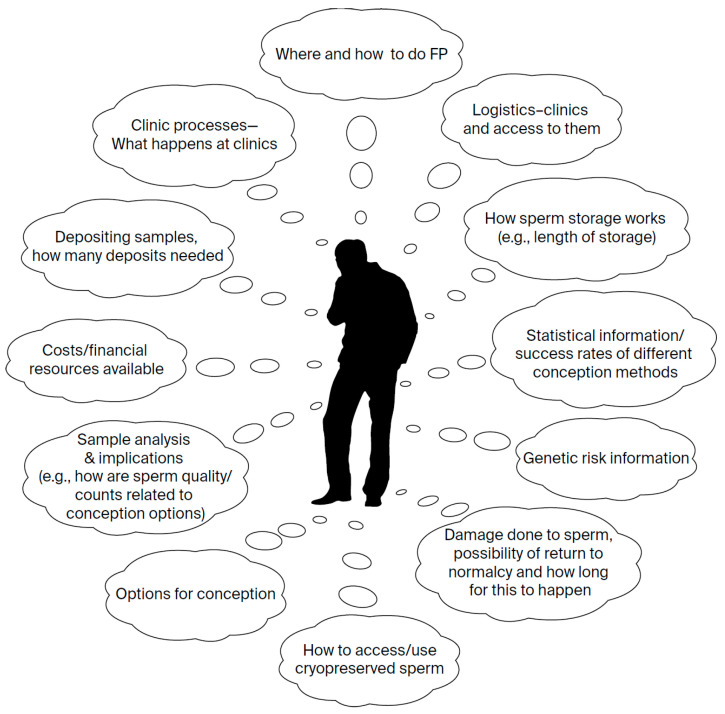
Specific informational needs about the fertility preservation process.

The men’s accounts also highlighted that their informational needs were not static; they needed different types of information at different points in their diagnosis–treatment–survivorship journey (Figure 1 and Figure 2 and Table 2). At diagnosis, they needed guidance on where and how to access FP, including clinic locations, referral pathways, wait times, and what to expect during clinic visits (e.g., sperm collection procedures and the number of samples or visits required). They also wanted information on storage—how long samples could be kept, storage costs, and available financial assistance. They described their need for information about sample analyses, including treatment-related damage, expected timeline for return to normal counts, and potential genetic risks. After treatment, the men needed guidance on how to access and use cryopreserved material, available options for conception, the relationship between sample quality and different conception methods, and statistical data on success rates.

The men reported that most of the written resources available were generic brochures, which did not give them a complete sense of ‘how it [FP] all worked’. One man recalled that he did not receive any specific FP materials but that he did come across ‘a few sentences in a section of my booklet that said fertility can be affected and to avoid trying to conceive within 6 months of chemo’ (P11). Even among those who had completed FP, an ongoing lack of clarity and uncertainty about the process persisted. The men’s accounts also suggest that clinics provided minimal information or guidance.

…[They] didn’t really talk to me about it. They just kind of did it, as like a procedure… “here’s the tube, fill it with this, come back, put your name on it and make sure it’s yours so we don’t mix them up”

(P8)

Not only did clinics not provide sufficient or detailed information, but one man reported that his request for results from his sperm analysis was denied (‘we don’t give it out, doctors have to request it’ (P2)).

Notably, although some men described FP information as ‘light’ or ‘pretty light,’ they also acknowledged that they did not always read materials provided. This reluctance appeared to stem partly from feelings of being overwhelmed and stressed during the diagnostic period, when they were given large amounts of complex information. The timing, pace and density of information, particularly for those who attended appointments alone, made it difficult to retain.

It was like okay, well, we’re going to move as quickly as we can through, what you got, what are the side effects, okay, there’s an option to bank sperm, here are all these people you are about to meet. It was like go, go, go. Within, like, less than a day…. I met one of my oncologists and while he was going through all the side effects of chemo, which he said we would start the next day, he did mention the probability of infertility. He may or may not have said you have the option to bank sperm, that I don’t remember…

(P7)

### 3.2. Emotional Support Needs

In addition to informational guidance, the men’s accounts highlighted their need for emotional support throughout the FP process (Table 3). Although FP was often perceived as a simple process (‘just getting a piece of paper and going to the clinic and just doing it’), the men’s experiences suggested otherwise. Some men were satisfied with the process; others reported feeling isolated and unsupported.

Here’s a piece of paper with a phone number, essentially, some colourful pamphlets, and “figure it out”. And that only gave us one option, I don’t even know if there’s more places you can bank in the city…. Nobody seemed to give a crap, like at the end of the day, it was up to [us] to try to figure out how to do this in a world where we didn’t even know anything about

(P2a)

They needed, this participant explained, more than encouragement to ‘just do it’. The men expressed a desire for meaningful counselling or coaching, particularly immediately after diagnosis and before treatment started. They emphasized the need for both immediate and sustained emotional support; as one participant said, ‘You need someone holding your hand through it’. Men also wanted practical step-by-step guidance immediately after diagnosis. One man described this as a ‘roadmap’, a structured pathway forward that would include ‘someone to come in after the doctor and say “okay, now you’ve had the diagnosis, here is where we go from here”’ (P2). Such support, he said, would have reduced his stress and anxiety during a time marked by shock, uncertainty, powerlessness and vulnerability. For some men, illness and the possibility of infertility were experienced as a threat to masculinity, which heightened this emotional burden. They emphasized the importance of having ongoing support throughout treatment, recovery and survivorship, for themselves and for their partners and families. The psychological impact on partners can be considerable, as one man noted, ‘It broke my wife’.

Participants also described FP decision-making as a directive rather than a collaborative process (‘it wasn’t like the doctor presented it as a question’). Rather than a shared process, the men’s accounts suggested they felt guided or pushed by physicians and family members. (‘I really think you should bank sperm’; ‘the surgeon really pushed, just like pushing me to do it’). Parental expectations, such as references to future grandchildren (wouldn’t it be nice if we were grandparents’) further added to this pressure. These decisions were made quickly, often without much time for reflection (‘I can’t say there is a decision…There wasn’t any time to ponder what to do, it’s just a matter of “okay, essentially where’s the closest place to get it done and how do we do it”’ (P2)). The urgency to begin treatment further reduced the men’s sense of agency and control.

… It felt like a ride… just caught up in this typhoon… kind of banging around… I felt like I was kind of just being strung along by this force of nature that was just pulling me and, I was kinda just going with it, just like, because my, my surgeon recommended [Cancer treatment centre] ‘cause that was the best option and I was like “oh okay, I will go with that one”, and my parents kind of suggested that I should look into fertility and get some sperm frozen and I was like “okay, I will do that” and I was, I was in a, I was very suggestable, which probably wasn’t the best place to be….

(P3)

In sum, the men’s experiences revealed a need for immediate and sustained emotional support. Their accounts reflected ambivalence about their FP outcomes. For some, there is lingering regret because they felt their choices were constrained, insufficiently informed, or overshadowed by other worries (‘I had lots of other things on my mind’).

### 3.3. Meeting Men’s Unmet Needs: Challenges and Tensions

**Figure 3:**
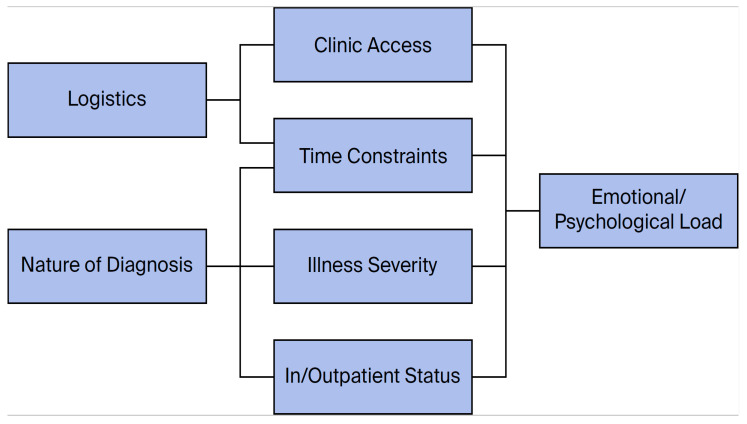
Challenges/tensions of meeting men’s unmet needs.

Multiple factors outside the men’s control shaped their FP outcomes (Figure 3). Logistical constraints, including compressed treatment timelines, difficulty accessing FP clinics and inpatients versus outpatients, together with diagnostic complexity (e.g., uncertainty and drawn-out processes) and illness severity (e.g., comorbidities such as edema), combined to make FP difficult to pursue. For some, psychological resistance to their diagnosis or to FP further shaped their choices and outcomes. The men’s reflections revealed a mix of ambivalence, distress, and diminished agency. One participant likened missing the opportunity to bank sperm to ‘rolling the dice’ on his future. Others who managed to bank sperm regarded it as a form of security or a contingency plan for potential infertility. Those unable to access FP expressed concerns about the long-term implications of infertility on their quality of life.

Our analysis suggests a complex interplay between cancer treatment, FP, and men’s informational and emotional support needs. The tension between urgent clinical priorities and the need for thoughtful, supported FP decision-making partly explains why men’s emotional and informational needs often remained unmet. As shown in Figure 3, logistical constraints—such as scheduling clinic appointments around accelerated treatment plans, accessing clinics and managing inpatient versus outpatient status—directly influenced when, how and how much information was given. Diagnostic uncertainty also shaped the timing and content of FP discussions. When diagnoses were uncertain or evolving, physicians delayed discussions because they believed they were premature and could exacerbate patients’ emotional distress (‘we are not there yet; I’m not going to give you too much information’). Illness severity further complicated the process. When men were severely unwell, FP was mentioned only briefly or was omitted altogether. Ultimately, physicians and patients tended to prioritize immediate treatment over sperm banking (‘the doctors didn’t want to wait, they wanted to start chemo right away’ (P6); ‘because I am super sick and if I [delayed treatment] I probably wouldn’t be around to use it’(P5)).

## 4. Discussion

Through this secondary analysis of qualitative data about male experiences with cancer and FP, we demonstrate that despite long-standing clinical guidelines and ongoing advocacy for FP, men at risk of treatment-related infertility continue to experience unmet support needs. While previous studies have identified gaps in information [27,28,29], this study extends that literature by mapping what men felt they needed and when. We highlight in detail the practical and psychosocial support they required to engage in knowledgeable FP decision-making across diagnosis, treatment and survivorship. They wanted guidance on accessing and navigating clinics, details about associated costs, clarity about specimen storage and instructions on how to access samples for future use.

The information provided was insufficient in content, scope, timing and delivery. Counselling at the point of diagnosis was brief, verbally communicated and poorly timed, which hindered comprehension and decision-making. Even those who had successfully banked sperm felt their knowledge remained limited. These findings build on and deepen insights from prior FP research [27,28,29]. Men also expressed a desire for FP counselling during clinic visits and throughout survivorship, with particular emphasis on understanding sperm quality, genetic risks, and pathways to conception. In their systematic review of 61 articles, Klinj et al. [26] identified three key support needs: (i) discussion of infertility risks with patients; (ii) consideration of the importance of future fertility; and (iii) provision of verbal and written patient-specific information. Although Klinj et al. [26] highlighted the need for more comprehensive information tailored to patients’ survivorship stage, they did not specify the types of information, and male survivors were underrepresented in the original studies.

These gaps in timing, content and delivery of FP information were closely linked to men’s experiences of FP decision-making and perceptions of empowerment. Within this complex interplay of factors, they experienced FP decisions as clinicians’ directives rather than as collaborative, deliberative choices. Although this ‘guidance-cooperation model’ [51] of doctor-patient communication is intended to shield patients from further cognitive overload, it can unintentionally reproduce paternalistic dynamics that could undermine patients’ sense of control. In our study, FP guidance was presented in ways that did not fully support men’s ability to make informed, autonomous decisions. Contextual factors such as the shock of a cancer diagnosis and the urgency to start treatment constrained the men’s ability to process and act on information. At these moments of heightened vulnerability, when they felt like they were ‘on a ride’ or were being ‘swept along’ by events, physicians’ and family members’ recommendations carried considerable weight. The erosion of autonomy has been associated with poorer psychological outcomes and diminished quality of life in cancer care contexts [28,52,53]. 

The men’s experiences of reduced agency also appeared to be intertwined with feelings of emotional strain. Determining the appropriate timing and amount of information at moments of vulnerability is challenging because too little or too much information can impede understanding. Providing detailed information about infertility risks and FP in tandem with discussions about diagnosis and treatment may increase psychological strain. Cancer patients often experience elevated levels of depression, anxiety and trauma at the time of diagnosis, and these feelings may persist throughout FP discussions and treatments [17]. When anxiety and fear are high, FP information may be experienced negatively [22,54], as overwhelming or distressing rather than as empowering.

The finding of men’s unmet informational and emotional support needs contrasts with Benedict’s [55] findings among female cancer patients, who did not pursue FP because they felt overwhelmed or were unaware of the option. These findings suggest that access to FP alone does not necessarily guarantee autonomous decisions. In our study, although men were offered and encouraged to bank sperm, the guidance provided was insufficient to support a strong sense of agency or control in their decision-making. Notably, under-utilization of sperm banking has often been attributed to lack of awareness [8,54,56], but our findings complicate this explanation. The men banked sperm despite having a limited understanding of the process, risks and long-term implications. This finding suggests how uptake of FP may create the appearance of well-informed decision-making while masking ongoing uncertainty and unmet guidance needs.

Beyond informational gaps, men described feelings of psychological strain when making FP decisions during diagnoses. Although sperm banking is often presented as a straightforward process, the men described feelings of vulnerability, fear and uncertainty, findings that are echoed in Ehrbar et al. [39], Tschudin and Bitzer [8], and Nahata et al. [20]. Our findings extend this work by highlighting how adult men also experience emotional challenges across both diagnosis and survivorship. Many of them expressed their desire for emotional support or counselling that began early in their FP journey and continued into survivorship. In the absence of such guidance, the men managed the emotional and logistical challenges of FP while simultaneously processing a cancer diagnosis. This finding illustrates how oncology systems routinely depend on patients and families to coordinate FP during periods of heightened stress and vulnerability.

Our findings also suggest that men’s experiences with FP were influenced by relational and culturally embedded norms of masculinity [57]. Social expectations that emphasize autonomy, agency, resilience, decisiveness, rationality, strength and virility [6,58] were often destabilized by the realities of cancer diagnosis and its treatment. Descriptions of FP and treatment suggested that these processes were happening to them rather than ones they actively navigated (‘it felt like a ride’, ‘being swept along’). Clinical directives about FP (‘you should really, really do it’), parental pressures (‘wouldn’t it be nice if we were grandparents’) and the urgency of treatment constrained deliberative decision-making and disrupted ideals of control.

Masculine norms, which prioritize decisiveness, control and responsibility, reframed FP from a clinical option into one tied to identity and moral responsibility. Decisions were influenced not solely by medical/clinical guidance but also by social expectations that men act responsibly to safeguard their potential for future fatherhood [6]. Research (e.g., Sahoo et al. [59]) shows that traditional masculine ideals shape how men experience threats to their reproductive potential and identity. Fatherhood is conceived as a life-stage responsibility and a marker of adult masculinity and virility [6,58]. As such, infertility may be experienced not merely as an iatrogenic effect of cancer treatment but also as a threat to men’s sense of self, social standing, and self-worth [10,58]. Within this context, FP carries dual meanings. It serves as ‘insurance’, a way to protect the possibility of future fatherhood, while enabling men to align with perceived societal and familial expectations of responsible masculinity [6,8,10,60,61]. 

These meanings are reinforced through interpersonal and clinical interactions. In interactions with parents and healthcare providers, fatherhood is often framed as an obligation. Likewise, authoritative clinical guidance presents FP as urgent and as a moral imperative (‘you’ll regret it’). Through these interactions, links between FP and responsibility, masculinity and future fatherhood are reinforced [61,62]. Masculine norms equate male identity with virility, which appears to heighten the pressure to bank sperm quickly before treatment begins. Familial involvement may further heighten men’s sense of obligation. Research shows that men frequently act with urgency in response to imminent treatment and social expectations, even when sperm quality may not be ideal [61,63]. Quick decisions to bank sperm, often made with inadequate information, reflect culturally valued traits of foresight, accountability, planning and decisiveness, sometimes performed at the expense of personal autonomy.

These same norms also appeared to shape how men navigated psychosocial support. Values of fortitude, self-reliance, and emotional control [57,58] influenced how men expressed and managed emotional turmoil. Although the men experienced fear, anxiety, and shock, they maintained composed exteriors and practical orientations. Framing FP as a technical task (‘just do it’) reinforced action-oriented mastery while minimizing its emotional significance. These norms muted the men’s need for emotional support and open expressions of worry about infertility because such displays of vulnerability are incompatible with ideals of masculinity [10,64]. Even as the men expressed a desire for more guidance or support (wanting someone to ‘hold their hand’ or offer a clear ‘roadmap’), they often hesitated to engage with informational materials or seek clarifications and instead accepted brief explanations from healthcare providers. This reluctance likely reflects tensions between vulnerability and expectations of competence rather than emotional disengagement.

At the same time, the men’s desire for detailed and staged FP information suggests attempts to reassert agency within these constraints. Admitting that ‘it’s hard when you don’t know what the questions are’ shows how uncertainty produces feelings of disempowerment. Seeking information appeared to function as a socially acceptable strategy for restoring feelings of competence, control and self-efficacy, without overtly challenging norms of self-reliance. These observations illustrate that men’s FP decision-making is not constrained by medical urgency alone but is also shaped by culturally embedded scripts of masculinity that influenced how they felt and acted within the context of these choices. Although most men banked sperm, many did so with limited understanding of the process, its risks, and long-term implications, which suggests that the uptake of FP may create the appearance of knowledgeable decisions while ongoing uncertainty and informational gaps persist. Thus, timely decisions to pursue FP may reflect a combination of factors such as clinical urgency, social expectations of masculinity, and partial or poorly timed information. These dynamics underscore that FP uptake alone does not guarantee empowered decision-making.

## 5. Strengths and Limitations

In this study, we used secondary qualitative analysis to explore the unmet informational and emotional needs of 12 men facing cancer treatment and infertility. Participants were chosen for their ability to provide in-depth accounts of their experiences [65]. Although we achieved informational redundancy—meaning no new information was forthcoming [66,67] —the small sample limits transferability to other settings. The COVID pandemic constrained our ability to achieve maximum variation and a larger sample. Nevertheless, these findings have naturalistic generalisability [67,68] or resonance with patients and clinicians in comparable contexts, as well as analytical generalisability [69], i.e., conceptualize men’s unmet needs as forms of social support within a social determinant of health framework for cancer survivorship.

This qualitative study contributes new insights to the oncology and FP literature. It maps men’s informational and emotional support needs across the diagnosis, treatment, and survivorship trajectory and specifies not just the types of information required but also when it is needed—an aspect often overlooked in current research. The findings highlight that partners/caregivers also experience emotional strain, which underscores the need to extend support to them. Additionally, the study illuminates men’s experience of vulnerability and emotional strain in the cancer and FP context. It shows how the seemingly routine procedures of sperm banking can be emotionally complex, particularly when decisions are shaped by clinician and situational pressures rather than patient autonomy. The study also demonstrates how systemic and logistical obstacles can restrict access to FP services, contribute to informational deficits and heighten emotional distress, which compromises agency and limits opportunities for empowered FP decision-making. By placing men’s accounts in a broader context, this study illustrates how such structural barriers can compromise agency and produce psychological harm by limiting opportunities for thoughtful FP decision-making.

These findings suggest several directions for future research and practice. We had initially theorized that FP experiences might vary across cancer types and contextual factors. Further research should investigate these potential differences. Our detailed mapping of men’s informational and emotional support needs provides a foundation for developing decision aids and practical tools to guide patients through the FP process. Clinically, the results highlight opportunities to embed structured support within oncology care, including counselling resources, patient navigators, fertility “champions,” and FP-trained advanced practice nurses who can coordinate care across the cancer and FP pathways. More broadly, the study underscores the need to recognize situational and structural barriers—not only information gaps—as potential contributors to the under-utilization of FP services.

Building on the work of Tschudin and Bitzer [8] and Hanna and Gough [58], who advocate for enhanced support of men’s decision-making in cancer and infertility contexts, our research highlights the necessity of directly addressing masculine norms when developing support interventions. This study suggests moving beyond a sole focus on psychological and biomedical models and integrating an understanding of the social and gendered dynamics that inform how men interpret infertility, their perceptions of responsibility, their feelings of agency and their willingness to seek help.

Our study also responds to calls (e.g., Orlic et al. [10]) for qualitative research using in-depth interviews to better understand men’s experiences of infertility in the context of cancer. By centering men’s own accounts, we showed how societal expectations of masculinity, fatherhood, and responsibility shape their emotional responses, decision-making and willingness to seek support. These findings have implications for clinical practice. They suggest the need for FP counselling that includes clear and timely information and that creates space for emotional expression. Such counselling should also recognize how men’s understandings of masculinity, including ideals of self-reliance, control, vulnerability and aspirations for future fatherhood, affect their engagement in fertility-related discussions. Viewed from this broader gendered social lens, unmet informational and support needs do not arise solely from failings in FP service delivery; rather, they emerge from the interaction between healthcare systems and prevailing gender norms, which together shape how men interpret, access, and respond to available support.

## 6. Conclusions

This study identifies important gaps in FP-related guidance and psychosocial support provided to men facing FP decisions during cancer treatment. Although FP decisions are treated as clinically straightforward, their experiences suggest that they are complex. The men were routinely required to make these decisions under conditions of emotional distress, diagnostic uncertainty and time pressures. At the same time, FP communications were often fragmented or insufficient to support knowledgeable decisions. FP counselling was narrowly focused on technical and biological details, with little attention paid to men’s (long-term) psychosocial concerns or their evolving informational needs. The current one-time FP conversation model is misaligned with these evolving needs. Our findings show that men needed not just more detailed information but also the right information at the right time—practical logistical guidance at diagnosis, clarity regarding storage during treatment and beyond, as well as meaningful counselling about reproductive options after treatment. Taken together, these findings suggest that FP decision-making is best understood as an ongoing process shaped by temporal, relational, and sociocultural factors rather than as a single clinical event.

This study reinforces previous research on the limited uptake of guideline-based FP counselling and extends existing evidence by identifying where, how and why FP-related decision support breaks down within oncology care. It adds nuance to FP decision-making research by illustrating how relationally and culturally embedded norms of masculinity, directive communication, emotional overload, diagnostic uncertainty and time pressures interact in ways that might negatively influence men’s well-being and shape their experiences of cancer and decisions. These findings support embedding FP within broader psychosocial and survivorship care, through clearer FP care pathways [26], tailored, stage-specific information, dedicated fertility navigators or onco-fertility coordinators and the integration of psychosocial supports that extend beyond diagnosis. Evidence from AYA oncology programmes shows that advanced practice nurses or social workers can play a pivotal role in facilitating knowledgeable choices, easing emotional burdens and advocating for patients around FP costs. The unmet needs articulated here are not an exhaustive list, but they highlight areas for further research and opportunities for developing targeted social and emotional supports for men and their families who may face fertility-threatening cancer treatments.

In sum, our findings show that men confronting cancer-related infertility require timely, tailored information and sustained psychosocial support—needs that are insufficiently addressed in current oncology care. Despite high rates of sperm banking, decisions were often made with limited understanding and diminished autonomy, which reflects broader structural gaps rather than individual failings. The findings also suggest that men’s seeming decisiveness may mask the effects of urgency, emotional overload and moral pressures that are embedded in clinical and social expectations. Addressing their needs will require integrated, patient-centred FP pathways that support both men and their families from diagnosis through survivorship. It also requires awareness that seemingly confident decisions may not be fully autonomous; they are being influenced by masculine ideals of identity and behaviour.

## Figures and Tables

**Table 1:** Study participant characteristics.

	Age at Diagnosis	Diagnosis	Relationship Status at Diagnosis	Children	FP Discussion and/or Referral	Who Referred/Raised	FP Follow Through
P1	17	testicular	single	0	Y	Surgeon, GP, oncologist	Y
P2	49	testicular	common-law	1	Y *	Oncologist	Y
P3	25	testicular	single	0	Y *	Mother	Y
P4	36	testicular	married	0	Y	Oncologist	Y
P5	29	Burkitt’s-stage 4	single	0	Y	Charge nurse	N
P6	31	lymphoma	single	0	Y	Oncologist	N
P7	37	testicular	single	0	Y	AYA programme director/hcp team	N
P8	31	testicular	married	0	Y	Urologist	Y
P9	34	testicular	married	0	Y	Urologist/oncologist	N
P10	29	testicular	married	1	Y	Urologist and resident	Y
P11	27	testicular	common-law	0	Y	Surgeon	Y
P12	22	lymphoma	single	0	Y	Hematologist/oncologist	Y
Average age	31						

Y *—Patient-initiated conversation. Y = Yes; N = No.

**Table 2:** Fertility preservation informational support needs across the cancer diagnosis, treatment and survivorship trajectory.

Informational Need	Pre-Treatment	During Treatment	Post-Treatment
FP process and logistics	Steps for banking: clinic locations, processes, timing, sample collection procedures-number of samples, timing between samples, ideal collection windows	Treatment impact clarity- clarification of whether further banking is possible	Accessing stored sperm; clinic pathways for use; disposal policies- options for unused sperm
Financial information	Costs, insurance coverage, storage fees, financial eligibility and aid	Ongoing cost considerations	Long-term storage fees; financial planning
Medical implications	Genetic risks; expected fertility impact of treatment	Updated expectations for fertility decline	Fertility recovery expectations; interpreting test results
Testing and monitoring	—	Whether monitoring is needed; coordination between medical teams re FP	Semen analysis interpretation; testing schedules
Reproductive planning	—	—	Conception options (IVF/IUI/ICSI); success probabilities

**Table 3:** Fertility preservation emotional support needs across the cancer diagnosis, treatment and survivorship trajectory.

Emotional Need	Pre-Treatment	During Treatment	Post-Treatment
Psychological support	Support for shock, distress, urgent decision-making; managing information overload	Coping with uncertainty, treatment stress, regret	Support for infertility grief, decisional regret and long-term adjustment
Navigation support	Help locating and accessing FP resources	Continued assistance with decision-making and support system navigation	Support managing long-term uncertainty and decisions
Family/partner support	Guidance for family involvement and emotional burden management	Communication and shared coping with and managing stress	Relationship and family planning support
Sense of control	Reassurance and clear guidance	Emotional reassurance during treatment	Support adapting to survivorship realities

## Data Availability

The data presented in this study are available on request from the corresponding author. The data are not publicly available due to privacy or ethical restrictions.
